# A Rare Case of Persistent ST-Elevation Myocardial Infarction Post-Tissue Plasminogen Activator With White Clot Extraction in a Middle-Aged Woman on Medroxyprogesterone Acetate

**DOI:** 10.7759/cureus.26628

**Published:** 2022-07-07

**Authors:** Syed S Fatmi, Paramjit Kaur, Emmanuel Tangco, Fadi Bader, Darius Aliabadi

**Affiliations:** 1 Internal Medicine, Southeast Health Medical Center, Dothan, USA; 2 Graduate Medical Education (GME) Internal Medicine, Southeast Health Medical Center, Dothan, USA; 3 Cardiology, Southeast Medical Center, Dothan, USA

**Keywords:** non-st elevation myocardial infraction, red clot, fibrin, pai-1, cardiac catheterization, depo-provera, tenectlapse, white clot, st elevation myocardial infarction

## Abstract

ST-elevation myocardial infarction (STEMI) occurs when vulnerable intravascular plaques rupture and produce eventual occlusion of the coronary circulation. With the increased prevalence of coronary artery disease, STEMIs and NSTEMIs are very well-studied and have generally been known to be caused by red and white thrombi, respectively. STEMIs have been more commonly associated with red clots, while NSTEMIs tend to be caused by white clots. Recent studies have also shown that a third of STEMIs are due to white clot formation, resulting in transmural infarction, most commonly seen at the coronary artery bifurcation. However, no cases of white clot STEMIs post-recombinant tissue plasminogen activator (rTPA) administration have been described in the literature. The data regarding the utility of rTPA in lysing white clots is limited, questioning the overall efficacy of rTPA with white clot lysis. This case report presents a patient on depot contraceptive who had a persistent STEMI despite rTPA administration and was found to have formed a white clot, which was extracted on thrombectomy. As this unique presentation and its associated risk factors are explored in the future, we hope that this case report contributes to the body of knowledge in the detection and management of white clot MIs in the context of rTPA efficacy.

## Introduction

An acute ST-elevation myocardial infarction (STEMI) results from an occlusive phenomenon in the coronary arteries that causes prolonged transmural myocardial infarction, resulting in eventual myocardial cell injury and/or necrosis [1]. The Fourth Universal Definition of Myocardial Infarction (MI) of 2018 ascertains that the presence of myocardial ischemic injury is confirmed with cardiac biomarkers such as high-sensitivity troponins and electrocardiogram (EKG) changes along with overall clinical presentation [2]. In general, an MI can be classified into five types based on etiology and pathogenesis [3]. Type 1 MI is due to an acute coronary atherothrombotic event with plaque rupture and vessel occlusion. Most patients who suffer MIs, whether STEMI or non-ST-elevation MI (NSTEMI), fall into this category.

The annual incidence of MIs in the United States is estimated to be around 805,000, with first-time MIs comprising approximately 75% of these cases [4]. As of data published in 2013, the annual incidence of fatal MI's is estimated to be 116,793 patients in the United States, with women comprising 43% of these cases and men, the slight majority at 57% [5]. Age is still an important non-modifiable risk factor with regard to MIs, with the average age of an MI being 65 years old for men and 72 years old for women [5]. STEMIs comprise around 38% of the annual incidence of MIs.

Recombinant tissue plasminogen activator (rTPA) is a temporizing intervention in the management of STEMIs. In the absence of a percutaneous coronary intervention (PCI) capable facility within a 120-minute radius of a patient’s initial point of care, rTPA is given prior to transfer to a PCI-capable facility for definitive management of a STEMI. rTPA is also a time-sensitive intervention, as current guidelines recommend that a healthcare provider at a non-PCI-capable facility decide within the first 30 minutes whether a patient with a STEMI will be loaded with rTPA or transferred to the nearest PCI-capable institution. However, there are studies that explore the prevalence of white thrombi and their relative resistance to thrombolysis. A study by Quadros et al. showed that up to a third of STEMIs were found to be caused by white clots [6]. Animal studies in rats and in-vivo studies have demonstrated that this type of clot has increased resistance to thrombolysis [7]. Plasminogen activator inhibitor (PAI-1), which is locally released by platelets and vascular endothelial cells, has been implicated in increasing platelet-rich clot resistance to thrombolysis [8,9]. Data are currently limited regarding the use of rTPA for these fibrin-rich platelet clots, and it has been suggested that determining thrombus consistency may provide useful prognostic information [10,11]. The following report is a case of a 41-year-old female who presented with a STEMI and, despite standard-of-care intervention with rTPA, was noted to have an intact platelet-rich white clot on emergent thrombectomy.

## Case presentation

A 41-year-old female presented to an outside facility around two hours after experiencing sudden-onset, severe, constant, centrally-located exertional chest pain radiating to her neck, with associated diaphoresis. The patient denied any associated dyspnea, lightheadedness, palpitations, or previous episodes of similar chest pain. Her past medical history was significant for obesity with a BMI of 49 kg/m^2^. Family history was non-significant for cardiac disease. Social history was significant for smoking around half a pack of cigarettes a day for the past 10 years. Prior to the presentation, the only medication that the patient was taking was intramuscular medroxyprogesterone acetate (Depo-Provera) every three months for contraception. On the day of her presentation, initial troponin at the outside facility was noted to be negative with EKG findings suggestive of ST-elevation MI. Prior to transfer to our institution, the patient was started on loading doses of aspirin and clopidogrel. The patient was also started on an intravenous heparin infusion. For ongoing chest pain, the patient also received sublingual nitroglycerin, which provided temporary relief. With the nearest PCI-capable facility being more than 120 minutes away, she was evaluated for any bleeding risk factors, and she was deemed safe to receive tenecteplase (TNKase) before being transferred to our facility for emergent cardiac catheterization and a higher level of care.

**Figure 1:**
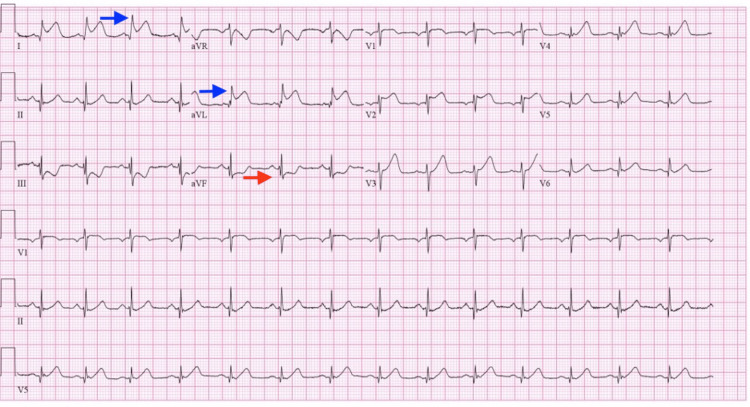
Electrocardiogram showing ST elevation myocardial infarction ST elevation noted in I, aVL illustrated by blue arrows with reciprocal changes noted by a red arrow.

**Figure 2:**
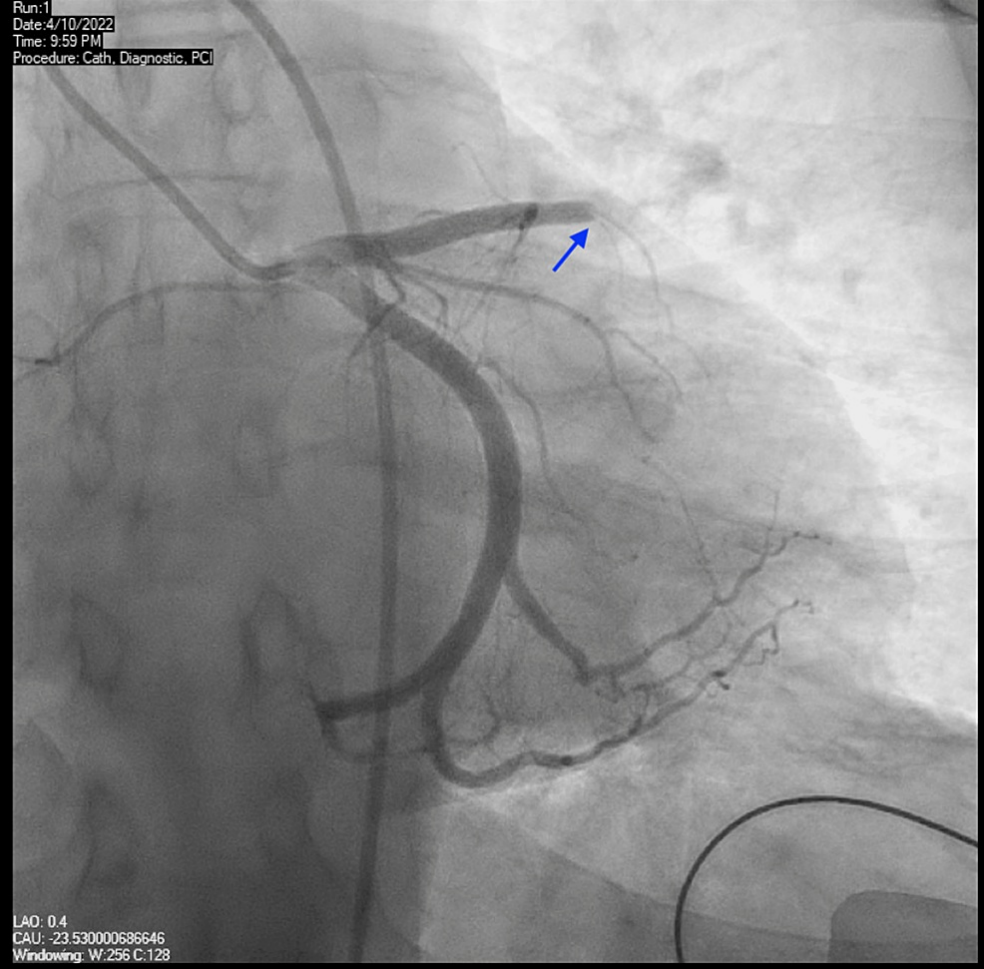
Cardiac catheterization images showing left anterior descending/diagonal Stenosis at the bifurcation caused by extracted white clot, blue arrow illustrating stenosis.

**Figure 3:**
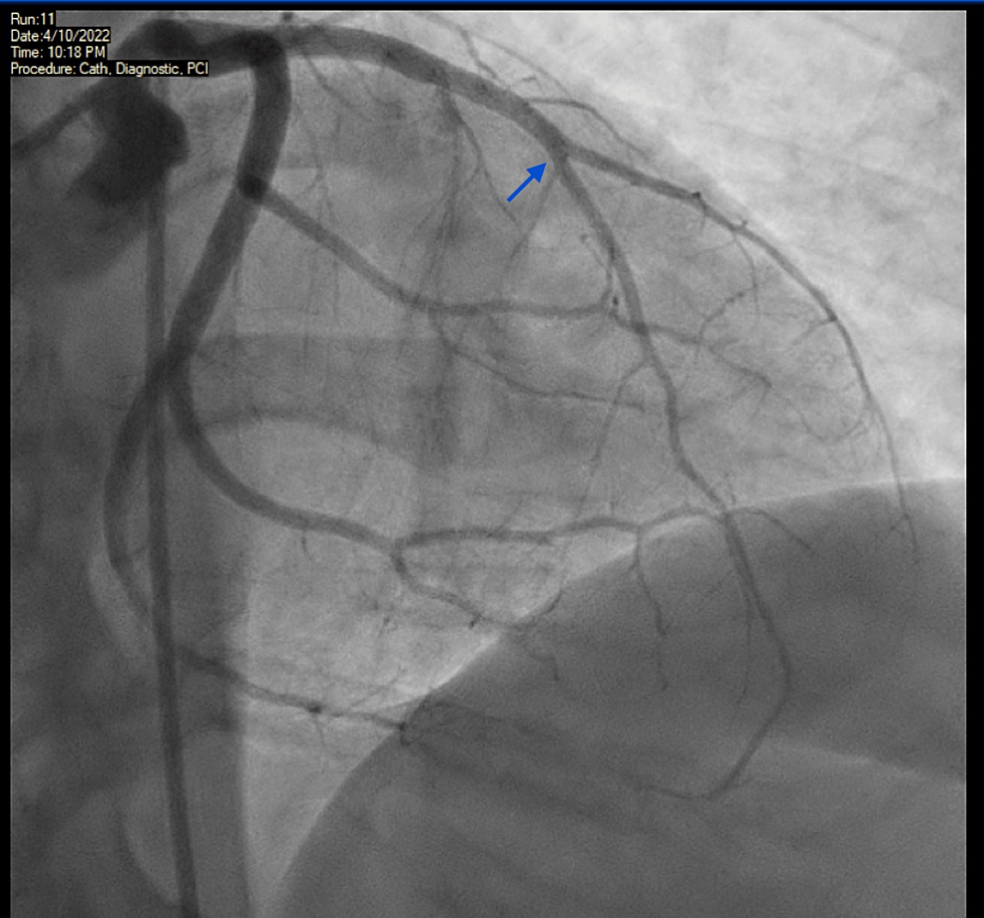
Cardiac catheterization images showing left anterior descending/diagonal Post white clot extraction image illustrated by blue arrow showing patent flow; TIMI grade 3 flow was restored to the LAD and its large diagonal branch.

Upon arrival at our emergency department, the patient was still experiencing chest pain and a repeat EKG at our facility revealed persistent ST-elevation in her anterolateral leads with reciprocal ST-depression in precordial leads as shown in Figure 1. In addition to her prior treatment, she was started on an intravenous nitroglycerin infusion as well. High sensitivity troponins peaked at 67,839 ng/L (reference normal range: <15 ng/L). A code STEMI was initiated and the patient was urgently taken to the cardiac catheterization laboratory. A coronary angiogram revealed a totally occluded left anterior descending artery (LAD)/diagonal at the midportion of the vessel at its takeoff and with the involvement of the principal diagonal branch (Figure 2). As shown in Figure 3, the left circumflex artery, two marginal branches, the left posterior descending artery, and the right coronary artery all appeared to have normal morphology. The thrombus was successfully extracted using export atherectomy. Following extraction, TIMI grade 3 flow was restored to the LAD and its large diagonal branch. Interestingly, following the clot extraction atherectomy, no plaque was noted in the vessels. It was also noted that the thrombus extracted was a white gelatinous clot.

After clot extraction arterectomy, the patient was transferred to the ICU for close monitoring for any reperfusion arrhythmias. A transthoracic echocardiogram was obtained during hospitalization to evaluate her left ventricle ejection fraction post-MI. Her echocardiogram revealed normal biventricular size and function with a global left ventricular ejection fraction of around 50%. She was discharged stable and chest pain-free on aspirin, clopidogrel, high-intensity statins, and bisoprolol. She was also counseled on smoking cessation and to use alternative methods of birth control other than Depo-Provera secondary to her increased risk of thromboembolism.

## Discussion

With this case report, we have described a STEMI case with white clot extraction. STEMIs are usually associated with red clots, whereas NSTEMIs have been known to be associated with white clots [12]. Despite receiving rTPA at the initial point-of-care, the patient still presented with an ongoing STEMI, with positive EKG findings, elevated high-sensitivity troponins, and active chest pain. During cardiac catheterization, a white clot was extracted, which is an uncommon finding in a patient who is post-rTPA administration. There was no coronary artery dissection noted during the extraction. The presumed mechanism of forming the white clot in this patient could have been due to erosion, as there was no other stenosis noted. The presumed mechanism for erosion could be secondary to endothelial injury and the formation of a platelet clot. White clots can be better evaluated with an intravascular ultrasound (IVUS), which was not done in this case due to the acuity of her STEMI presentation.

Studies have shown that rTPA works on blood clots by attaching to the fibrin on the clot surface, which activates the bound plasminogen, causing plasmin to be cleaved from the plasminogen and subsequently resulting in plasmin breaking up the fibrin molecule and dissolving the clot. This mechanism of action, however, cannot be postulated for white clots. It is possible that TPA works by a similar mechanism on white clots, however, TPA did not dissolve the white clot in our patient. It is unclear why rTPA has decreased efficacy with white clots, but it is concerning as, although not the majority, some STEMI patients have white clots and do end up receiving rTPA in non-PCI-capable facilities.

White clots form by erosion, and there are multiple mechanisms that can contribute to erosion. As described in our case, the patient developed a platelet clot at the bifurcation of the LAD with other vessels taking off. Endothelial erosion tends to occur on thick, capped atherosclerotic regions, which may or may not be associated with inflammation. The caveat was that our subject had no atherosclerosis, which is one of the main risk factors for erosion. Endothelial erosions can be better evaluated by IVUS, which was not performed in this case due to the acuity and urgency of the patient’s STEMI presentations. However, this represents an avenue of future intervention in cases presenting with similar, yet subacute presentation.

In this case report, our patient did not have any significant underlying cardiovascular disease. The possible mechanism of action behind her MI could be possible endothelial erosion, as previous literature has shown that female sex and smoking are predisposing strong risk factors. Her depot hormone therapy might have compounded her risk for plaque erosion as well, which also needs to be taken into consideration as the patient was not noted to have any other abnormalities in her labs that would represent a modifiable risk factor for early onset coronary artery disease.

## Conclusions

ST-elevation myocardial infarction is associated with transmural infarction and has traditionally been associated with a red clot in the majority of cases. We have described an extremely rare case of the white clot with STEMI presentation in a patient who had already received tPA prior to presentation. This case is rather unique because of the age of the patient, lack of non-modifiable risk factors, and the patient being on depot contraceptive therapy. Although coronary artery clots are more common at the bifurcation of coronary arteries, as was the case with this patient, the presentation of white clots in middle-aged women post-rTPA is extremely rare. We hope that this case report will help identify more cases in the future, so further research can be done to understand the pathophysiology of persistent white clots post-rTPA. It will also create awareness and give more insight into any associations that might or might not exist with cases of STEMI and the use of depot forms of contraception.
